# The Oxidoreductase DsbA1 negatively influences 2,4-diacetylphloroglucinol biosynthesis by interfering the function of Gcd in *Pseudomonas fluorescens* 2P24

**DOI:** 10.1186/s12866-020-1714-1

**Published:** 2020-02-24

**Authors:** Bo Zhang, Hui Zhao, Xiaogang Wu, Li-Qun Zhang

**Affiliations:** 1 College of Agriculture, Guangxi University, Nanning, 530004 China; 2 College of Plant Protection, China Agricultural University, Beijing, 100193 China

**Keywords:** *Pseudomonas fluorescens*, 2,4-DAPG, Disulfide bond, Oxidoreductase DsbA1, Glucose dehydrogenase Gcd

## Abstract

**Background:**

The polyketide antibiotic 2,4-diacetylphloroglucinol (2,4-DAPG), produced by *Pseudomonas fluorescens* 2P24, is positively regulated by the GacS-GacA two-component system.

**Results:**

Here we reported on the characterization of DsbA1 (disulfide oxidoreductase) as novel regulator of biocontrol activity in *P. fluorescens*. Our data showed that mutation of *dsbA1* caused the accumulation of 2,4-DAPG in a GacA-independent manner. Further analysis indicated that DsbA1 interacts with membrane-bound glucose dehydrogenase Gcd, which positively regulates the production of 2,4-DAPG. Mutation of cysteine (C)-235, C275, and C578 of Gcd, significantly reduced the interaction with DsbA1, enhanced the activity of Gcd and increased 2,4-DAPG production.

**Conclusions:**

Our results suggest that DsbA1 regulates the 2,4-DAPG concentration via fine-tuning the function of Gcd in *P. fluorescens* 2P24.

## Background

Among plant growth-promoting rhizobacteria (PGPR), fluorescent pseudomonads have received particular attention because of their ability to aggressively colonize in the rhizosphere, induce systemic resistance in plants, and protect plants against phytopathogens [1]. Many *Pseudomonas* species are well-studied PGPRs and secrete a battery of antimicrobial metabolites, including 2,4-diacetyphloroglucinol (2,4-DAPG), phenazines, pyoluteorin, pyrrolnitrin, hydrogen cyanide, and nonribosomal peptides [2]. Particularly, 2,4-DAPG has been extensively investigated as a key determinant in *Pseudomonas fluorescens*’s biocontrol activity against the wheat take-all decline caused by *Gaeumannomyces graminis* var. *tritici* [3, 4].

2,4-DAPG is a phloroglucinol derivative and is synthesized by the *phlACBD* locus transcribed as a single operon [5]. The *phlD* gene encodes a type III polyketide synthases and is required for the synthesis of phloroglucinol (PG) from malonyl-coezyme A [6]. The *phlA*, *phlC* and *phlB* genes together mediate the conversion of PG to monoacetylphloroglucinol (MAPG) and of MAPG to 2,4-DAPG [7]. Biosynthesis of 2,4-DAPG is regulated by multiple genetic elements. The *phlE* gene, located immediately downstream of the *phlABCD* locus, encodes a putative permease that serves as an export protein. PhlE is believed to secrete toxic intermediates of 2,4-DAPG degradation out of the cells [8]. The divergently transcribed *phlF* gene, located adjacent to *phlA*, encodes a pathway-specific transcriptional repressor. Repression by PhlF is achieved via its interaction with an inverted repeated sequence, *phO,* located upstream of the *phlA* transcription start site [9]. Finally, *phlG* encodes a hydrolase that specifically degrades 2,4-DAPG to less toxic MAPG and acetate [10]. Recent studies showed that another pathway-specific transcriptional repressor, PhlH, modulates 2,4-DAPG levels by controlling the expression of the *phlG* gene by sensing the concentration of 2,4-DAPG and MAPG in cells [11].

In addition, the biosynthesis of 2,4-DAPG is influenced by many global regulatory elements in response to the physiological status of the bacterial cell or environmental factors. The Gac/Rsm signal transduction system positively regulates the production of 2,4-DAPG and other secondary metabolites by fine-turning the output of the Rsm system [12]. Many sigma factors, such as RpoD, RpoS, and RpoN, may also profoundly influence 2,4-DAPG synthesis in response to environmental cues [13–15]. The glucose-inhibited division protein A (GidA) and tRNA modification GTPase (TrmE) inhibit the synthesis of PG and then decrease the accumulation of 2,4-DAPG in cells [16]. Besides global regulators, different carbon and nitrogen sources, metal ions, and metabolites secreted by bacteria and pathogenic fungi may modulate 2,4-DAPG production. For instance, 2,4-DAPG biosynthesis in *P. fluorescens* 2P24 is negatively affected by sucrose, but positively regulated by glucose [17].

*P. fluorescens* 2P24 is an effective biocontrol agent of soilborne plant diseases caused by phytopathogens [18]. The production of 2,4-DAPG is a crucial biocontrol determinant and is involved a complex regulatory network in this strain [4]. In the present study, we demonstrated that the protein disulfide oxidoreductase *dsbA1* gene negatively regulated the production of 2,4-DAPG by fine-tuning the function of glucose dehydrogenase (Gcd) in *P. fluorescens* 2P24. Further analysis indicated that three cysteine residues, C235, C275, and C578 in Gcd were required for the interaction between DsbA1 and Gcd. These findings provide a new insight into 2,4-DAPG production in which DsbA1 influences the production of 2,4-DAPG via Gcd at the post-transcriptional level.

## Results

### The production of 2,4-DAPG was negatively regulated by DsbA1

In an approach to identify novel regulators of 2,4-diacetyphloroglucinol (2,4-DAPG) production in *P. fluorescens*, the *gacA* mutant strain PM203 was subjected to a random Tn*5* insertion mutagenesis. Among the 5000 mutants tested, four mutants exhibited the antifungal activity against plant pathogen *Rhizoctonia solani* compared with the *gacA* mutant (Additional file 2: Table S2). Sequence analysis showed that in one of the mutants, X-2, the transposon was inserted into the *dsbA1* gene. The *dsbA* gene encodes a major periplasmic disulfide-bond-forming protein. An in silico analysis revealed two genes in *P. fluorescens* 2P24 genome (accession number CP025542) encoding DsbA family proteins (DsbA1 [C0J56_00210] and DsbA2 [C0J56_08555]), which have 28 and 13% amino acid sequence identity with DsbA from *E. coli*, respectively. In addition, two genes encoding proteins homologous to DsbB (DsbB1 [C0J56_24475] and DsbB2 [C0J56_29125]), which is required for reoxidizing DsbA’s cysteines to regenerate its activity, are found in the 2P24 genome. DsbB1 and DsbB2 of *P. fluorescens* 2P24 share 29 and 26% identity with *E. coli* DsbB, respectively.

**Fig. 1:**
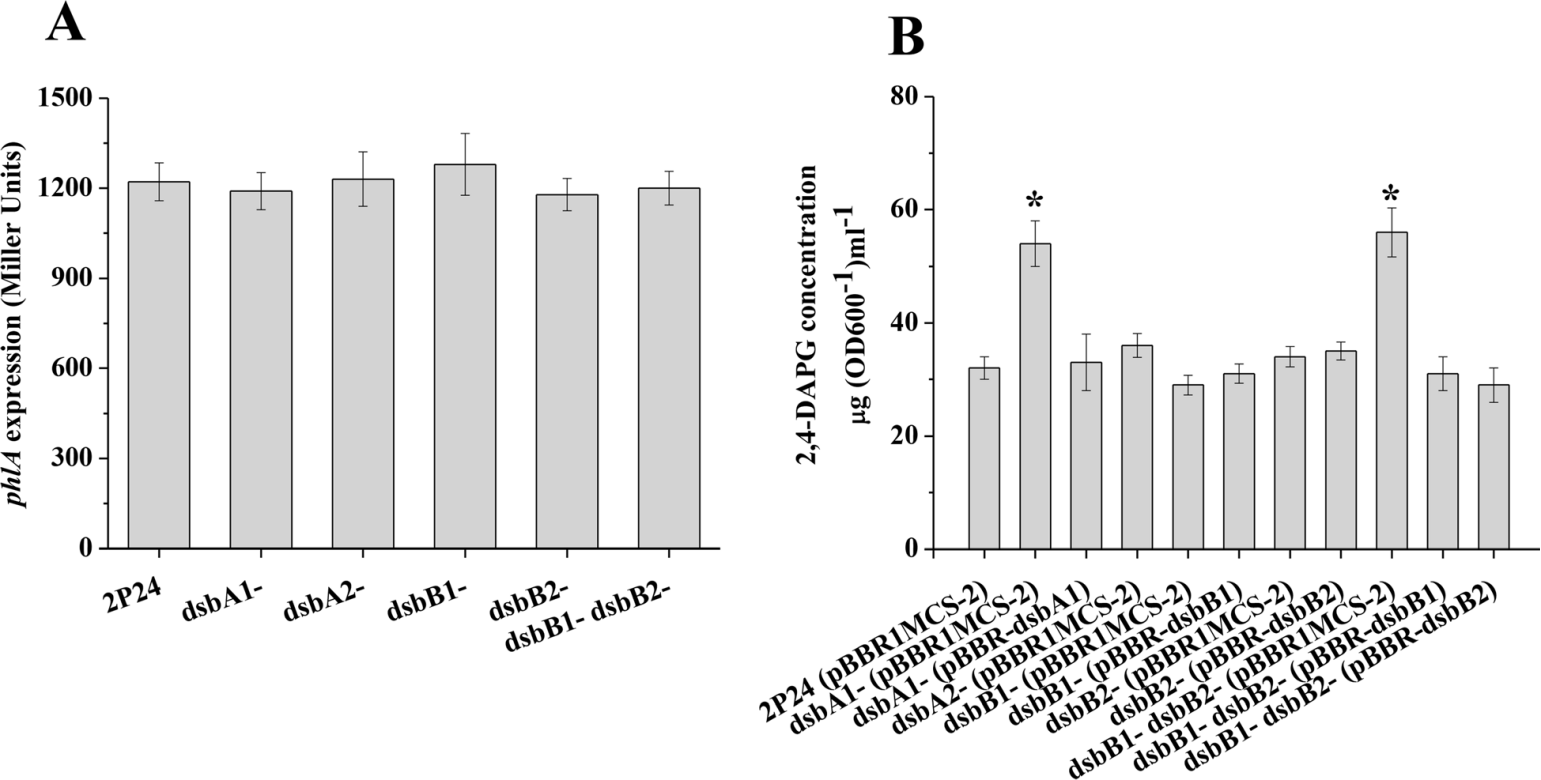
Effect of *dsbA1*, *dsbA2*, *dsbB1*, and *dsbB2* mutations on the expression of *phlA* and 2,4-DAPG production. **a** The plasmid-borne *phlA*′-′*lacZ* fusion on p6013-phlA was determined in *P. fluorescens* 2P24 and its derivatives. **b** HPLC analysis of 2,4-DAPG production by strain 2P24 and its derivatives in KB medium with 2% glucose. The experiment was performed in triplicate, and the mean values ± SD are indicated. * indicates *P* < 0.05

DsbA family proteins are involved in the oxidative folding of various proteins [19]. To determine whether DsbA1 regulates 2,4-DAPG production, we checked the effect of Dsb proteins on the expression of *phlA* in strain 2P24. Translation fusion assays showed that mutation in *dsbA1*, *dsbA2*, *dsbB1*, or *dsbB2* could not influence the *phlA′-′lacZ* expression (Fig. 1a). Whereas HPLC analysis indicated that more 2,4-DAPG was produced in the *dsbA1* and the *dsbB1 dsbB2* double mutant than in the wild type (Fig. 1b). By contrast the *dsbA2* and the single *dsbB* mutants produced similar amounts to strain 2P24 (Fig. 1). Introduction of the plasmid-borne *dsbA1* gene in the *dsbA1* mutant restored 2,4-DAPG produced to the level of wild-type strain. Similarly, the introduction of the plasmid-borne *dsbB1* gene or *dsbB2* gene into the *dsbB1 dsbB2* double mutant restored the production of 2,4-DAPG (Fig. 1b). These results indicated that DsbA1, DsbB1, and DsbB2, but not DsbA2, act as negative regulatory elements in the synthesis of 2,4-DAPG.

### DsbA1 regulates the production of 2,4-DAPG in a Gac/Rsm-independent manner

**Fig. 2:**
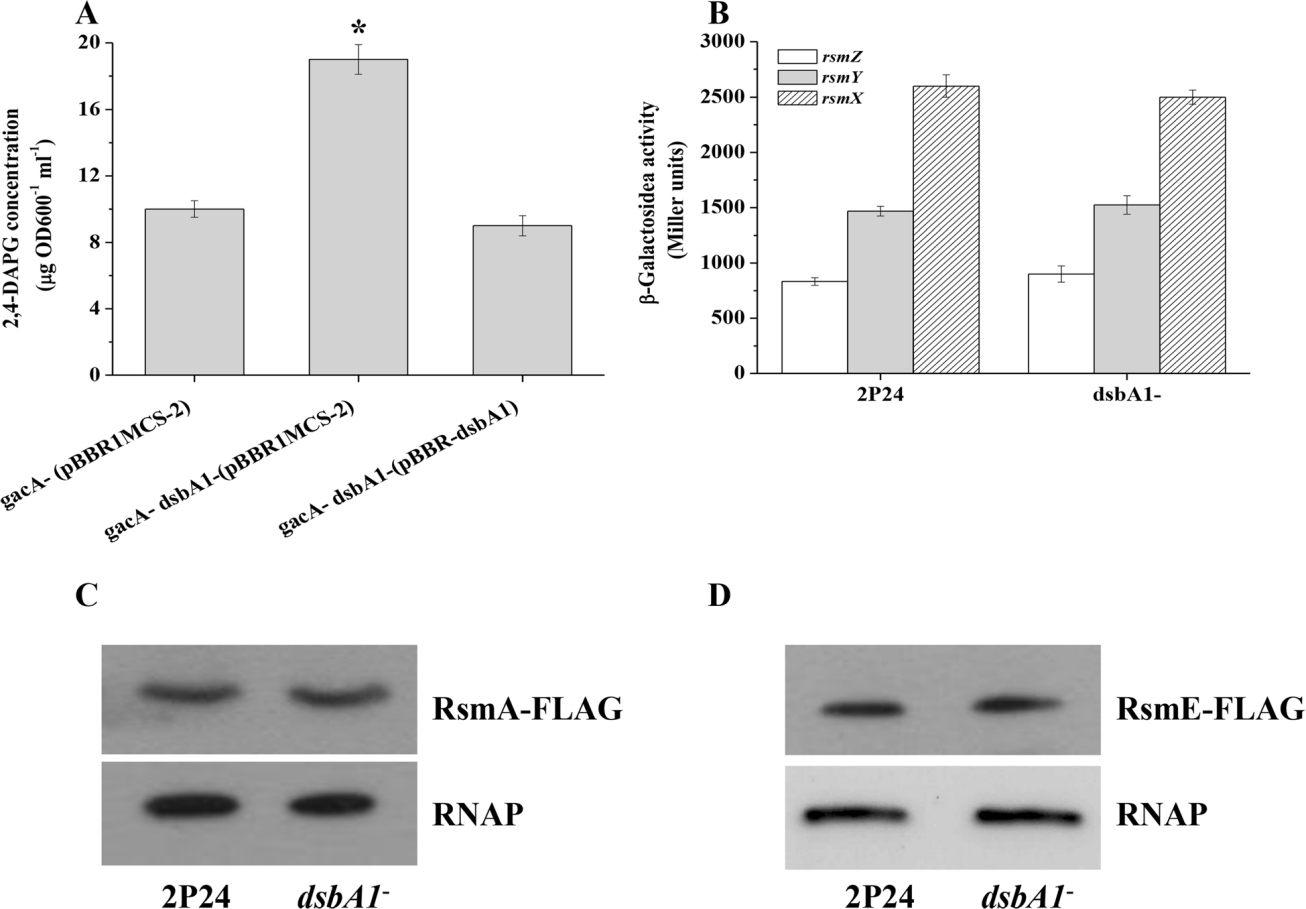
DsbA1 regulated 2,4-DAPG production in a Gac/Rsm-independent manner. **a** HPLC analysis of 2,4-DAPG production by the *gacA* mutant and the *gacA dsbA1* double mutant in KB medium with 2% glucose. **b** The expression of *rsmZ*-*lacZ*, *rsmY*-*lacZ*, and *rsmX*-*lacZ* transcriptional fusion was determined in *P. fluorescens* 2P24 and its *dsbA1* mutant, respectively. The experiments were performed in triplicate, and the mean values ± SD are indicated. * indicates *P* < 0.05. Western blot analysis was performed to detect RsmA-FLAG (**c**) and RsmE-FLAG (**d**). Three independent experiments were performed and a representative blot was shown

Our results showed that the production of 2,4-DAPG was significantly increased in the mutant X-2. To verify this phenotype, we further constructed the *dsbA1 gacA* mutant and tested its effect on 2,4-DAPG production. Compared to the *gacA* mutant, 2,4-DAPG production was significantly increased in the *dsbA1 gacA* double mutant. This could be complemented by introducing a copy of wild-type *dsbA1* on the plasmid pBBR-dsbA1 (Fig. 2a).

The GacS/GacA system exerts its function via the small regulatory RNA (sRNA) RsmX, RsmY, and RsmZ to sequester the CsrA/RsmA family proteins RsmA and RsmE [1]. To determine whether DsbA1 negatively regulated 2,4-DAPG production via sRNAs or RsmA and RsmE proteins, we compared the expression of these regulatory elements in wild-type and the *dsbA1* mutant. Similar to the wild-type, mutation of *dsbA1* could not change *rsmX*, *rsmY*, and *rsmZ* genes expression (Fig. 2b). Western blot assay further showed that similar levels of the RsmA and RsmE proteins were observed between the *dsbA1* mutant and the wild-type strain 2P24 (Fig. 2c & d). Taken together, these results suggested that DsbA1 affects the production of 2,4-DAPG in a Gac/Rsm-independent manner in *P. fluorescens*.

### The C235, C275, and C578 cysteine residues of Gcd are essential for the interaction of DsbA1 in vivo

**Fig. 3:**
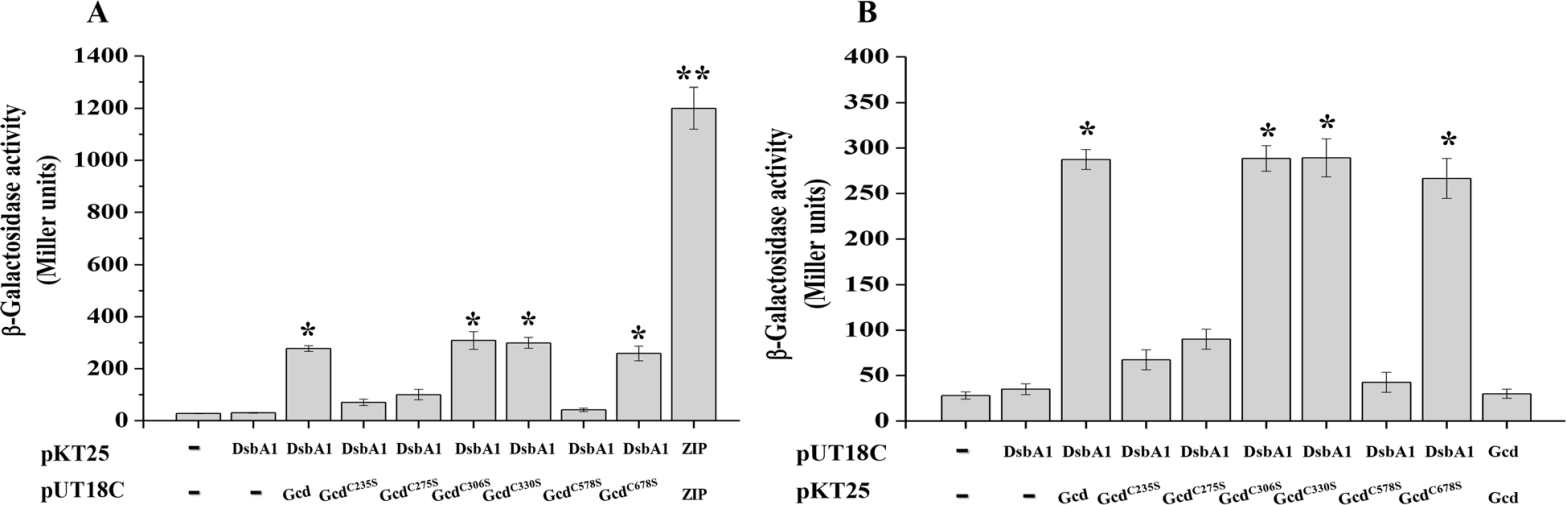
The interaction of DsbA1 with Gcd and its derivatives in vivo. DsbA1, Gcd and its derivatives were fused with the T25 (**a**) and T18 (**b**) domains of CyaA from *Bordetella pertussis*, respectively, and the T25, T18 fusion pairs were transformed into *E. coli* BTH101 cells. Cultures were grown at 30 °C for 8 h and the β-galactosidase activities were then measured using Miller method [23]. The experiments were performed in triplicate, and the mean values ± SD are indicated. * indicates *P* < 0.05

The function of DsbA1 is to form disulfide bonds between consecutive cysteine residues in its target proteins, we thus hypothesized that DsbA1 might catalyze the formation of disulfide bonds on a regulator of 2,4-DAPG production, which is localized on cell membrane or in the periplasmic space. Several proteins containing cysteine residues, including the pathway-specific transcriptional repressor PhlF [20], outer membrane protein OprF [21], and glucose dehydrogenase Gcd [22] were selected for a bacterial two-hybrid system with DsbA1. A strong interaction was only detected between DsbA1 and Gcd (Fig. 3 & Additional file 1: Figure S1), a glucose dehydrogenase that is required for the conversion of glucose to gluconic acid [24]. Analysis using PredictProtein (http://www.predictprotein.org) suggested that Gcd is a transmembrane protein with six cysteine residues C235, C275, C306, C330, C578, and C678 in the periplasmic space. Individual mutagenesis of these periplasmic cysteine residues into serine revealed the critical roles of C235, C275, and C578 in the interaction between Gcd and DsbA1 (Fig. 3). In addition, we noticed that the fusions containing only Gcd were unable to reconstitute significant β-galactosidase activities when coexpressed in *E. coli*, suggesting that Gcd exerts its biological function as a monomer (Fig. 3b).

### DsbA1 represses 2,4-DAPG production in a Gcd-dependent manner

**Fig. 4:**
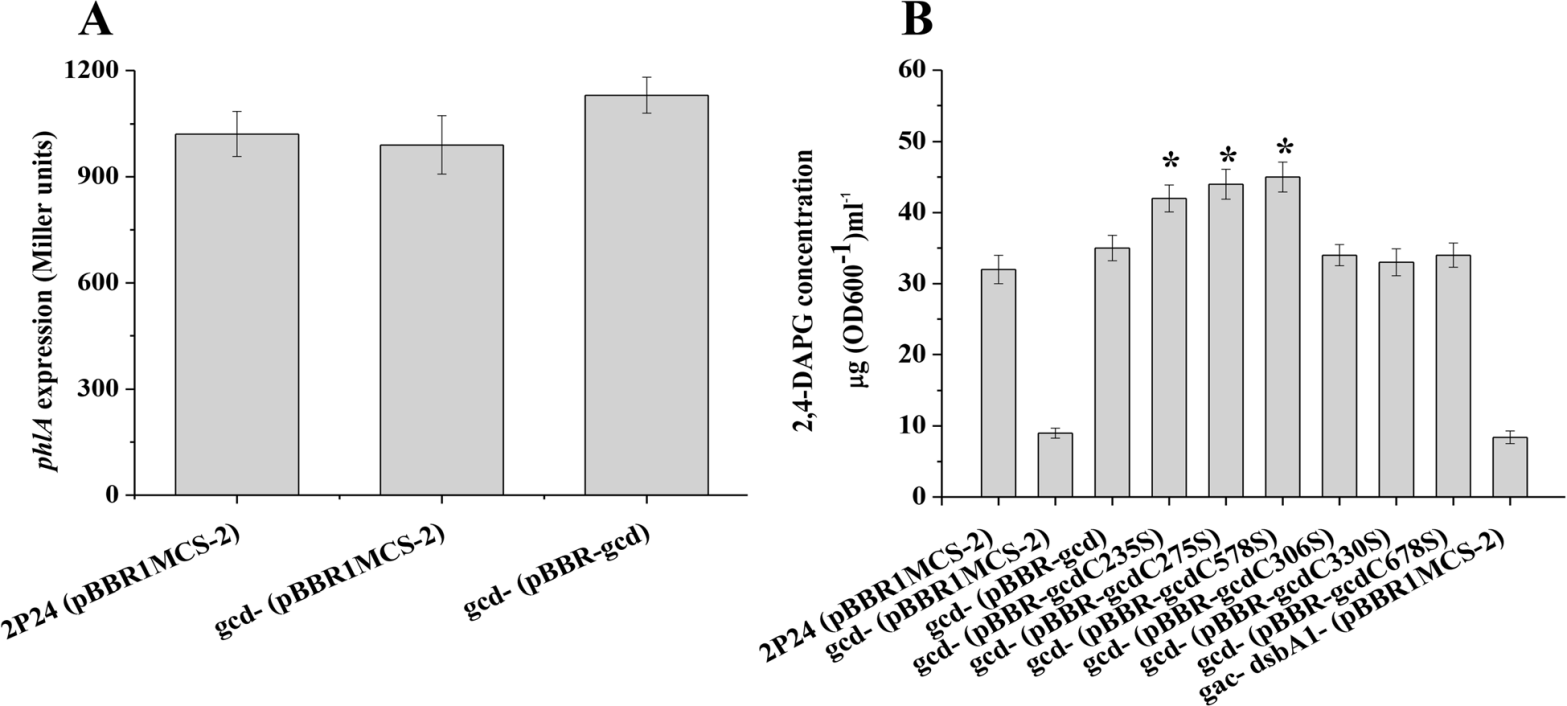
Regulation of the expression of the *phlA* gene and the production of 2,4-DAPG by Gcd. **a** The plasmid-borne *phlA′-′lacZ* fusion on p6013-phlA was determined in *P. fluorescens* 2P24 and the *gcd* mutant. **b** Biosynthesis of 2,4-DAPG in strain 2P24 and its *gcd* mutant was assayed by HPLC. All experiments were performed in triplicate, and the mean values ± SD are indicated. * indicates *P* < 0.05

The direct interaction between DsbA1 and Gcd raised the possibility that DsbA1 might regulate the production of 2,4-DAPG via Gcd. We thus examined the effect of Gcd on the production of 2,4-DAPG. β-Galactosidase reporter assays showed that the translation *phlA′-′lacZ* fusion did not differ significantly in the *gcd* mutant from that in the wild-type (Fig. 4a), but 2,4-DAPG production was 3-fold lower than that in the wild-type 2P24. The plasmid-borne *gcd* gene restored 2,4-DAPG production in the *gcd* mutant, indicating the positive regulation of Gcd on 2,4-DAPG production (Fig. 4b). Furthermore, we observed that repression of 2,4-DAPG production in the *dsbA1* mutant was abolished by in-frame deletion of *gcd*, indicating that DsbA1-mediated repression of 2,4-DAPG is Gcd-dependent (Fig. 4b).

**Fig. 5:**
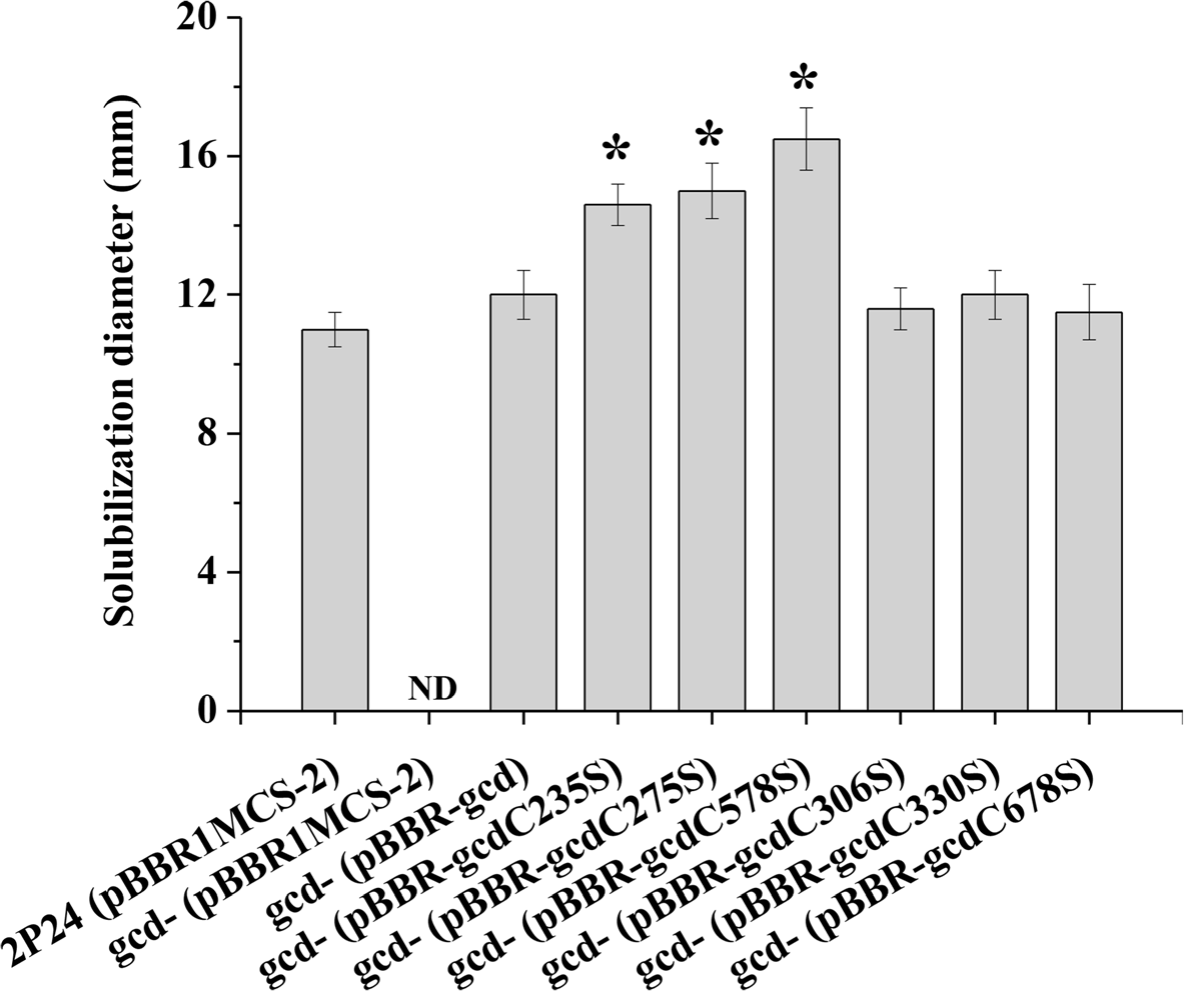
The effect of *gcd* on phosphate solubilization. The bacterial strains were grown on NBRIP agar plates and the solubilization of tricalcium phosphate resulted in the formation of cleared zones after 20 d of incubation at 28 °C. the experiment was performed in triplicate, and the mean values ± SD are indicated. * indicates *P* < 0.05

Given that DsbA1 interacts with Gcd and that DsbA1 negatively, but Gcd positively influences the concentration of 2,4-DAPG, we hypothesized that mutation in *dsbA1* would improve the activity of Gcd. To test this hypothesis, we checked the concentration of 2,4-DAPG in the Gcd cysteine mutations. Interestingly, the C235S, C275S, and C578S mutations increased the concentration of 2,4-DAPG. Whereas the C306S, C330S, and C678S mutations could not change the concentration of 2,4-DAPG in the cells of *P. fluorescens* (Fig. 4b). Gcd catalyzes the conversion of glucose to gluconic acid, which is efficient to solubilize mineral phosphate on NBRIP agar plates. The halo size produced by the wild-type 2P24 on NBRIP plate was about 11 mm in diameter, whereas those formed by the C235S, C275S, and C578S mutations were about 15 mm, indicating that mutations of C235, C275, and C578 improved the function of Gcd (Fig. 5).

### The effect of *dsbA1*, *dsbB1*, and *dsbB2* genes on the swimming motility and twitching motility

**Fig. 6:**
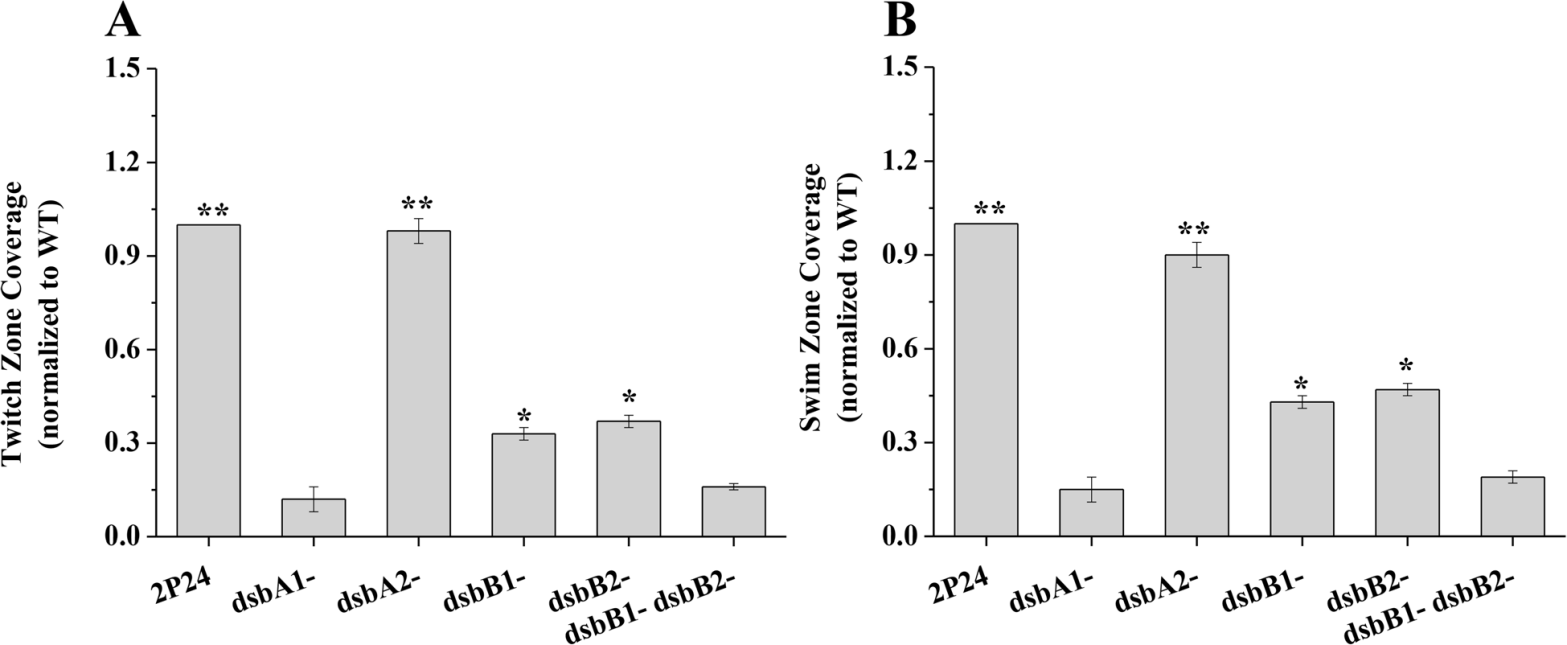
The effect of *dsbA1*, *dsbA2*, *dsbB1*, and *dsbB2* on the cell motility of *P. fluorescens* 2P24. The area covered by the twitching motility zone (**a**) or swimming motility zone (**b**) was normalized to that of the wild-type strain, which was set to a value of 1, for ease of comparison. The experiment was performed in triplicate, and the mean values ± SD are indicated. * indicates *P* < 0.05, and ** indicates *P* < 0.01

Previous data showed that DsbA is essential for *E. coli* cell motility [25]. To verify the role of DsbA and DsbB proteins in cell motility, we examined the motility of strain 2P24 and its derivatives. The results showed that the *dsbA1* mutant was defective in both swimming and twitching motilities, however, the *dsbA2* mutant had a normal phenotype (Fig. 6). Although the single *dsbB* mutants exhibited significant defects in swimming and twitching motilities, disruption of both the *dsbB1* and *dsbB2* genes resulted in severe defects in cell motilities (Fig. 6). These results indicated DsbA1, DsbB1, and DsbB2 are essential for *P. fluorescens* 2P24 cell motility.

## Discussion

Disulfide bond formation is essential for the function or stability of many extra-cytoplasmic and secreted proteins that contain more than one cysteine residue [19]. In many Gram-negative bacteria, incorporation of disulfide bonds takes place in the periplasmic space and is orchestrated by the DsbA/DsbB oxidation pathway [26]. In this study, we identified that DsbA regulated the production of 2,4-DAPG in *P. fluoresces* 2P24 (Fig. 1). Using Illumina Solexa-based whole-genome sequencing, we obtained the whole-genome sequence of strain 2P24 (accession number: CP025542) and subsequently found that the genome of strain 2P24 has two *dsbA* gene homologs (*dsbA1* and *dsbA2*) and two *dsbB* homologs (*dsbB1* and *dsbB2*). Our data indicated that *dsbA1* and both *dsbB* genes were required for the production of 2,4-DAPG, and the cell’s twitching and swimming motility, suggesting that DsbB1 and DsbB2 together are involved in recycling reduced DsbA1 to the active oxidized state in strain 2P24. Similarly, in *P. aeruginosa*, it has been suggested that the PaDsbB1 and PaDsbB2 control the redox state of PaDsbA1, because the *PadsbB1B2* double mutant and the *PadsbA1* mutant showed similar phenotypes [27]. Furthermore, two *dsbA* gene homologs were found in the genome of plant pathogen *Xanthomonas campestris* pv. *campestris* (*Xcc*) and mutation in both of *dsbA* genes exhibited attenuation in virulence and hypersensitive response, indicating that both *dsbA* genes are required for pathogenesis process [28]. In contrast to DsbA from *Xcc*, DsbA2 of *P. fluorescens* and *P. aeruginosa* belongs to a different subclass of DsbA proteins [27]. This protein possesses four conserved cysteine residues, and an invariant threonine residue preceding the *cis*-proline found in proteins with a thioredoxin (Trx) fold. This functional differentiation of DsbA could contribute to the survival of *Pseudomonas* sp. in specific habitats.

The GacS/GacA system plays a critical role in the production of 2,4-DAPG, and the production of 2,4-DAPG was severely reduced in the *gacA* mutant [29]. However, mutation of the *dsbA1* gene significantly increased the production of 2,4-DAPG in the *gacA* mutant, and the expression of sRNA and protein levels of RsmA and RsmE were not changed in the *dsbA1* mutant compared with that of wild-type (Fig. 2 & Additional file 1: Figure S1). These data suggested that DsbA1 regulates the production of 2,4-DAPG independent of the Gac/Rsm signaling pathway.

Our discovery of the interaction between DsbA1 and Gcd revealed a new regulatory pathway for fine-tuning the production of 2,4-DAPG in *P. fluorescens* 2P24. Gcd is a positive factor for 2,4-DAPG production in strain 2P24. Our data suggested mutation of *dsbA1* improved the function of Gcd and then enhanced 2,4-DAPG production. The Gcd protein contains six cysteine residues located in the periplasmic space. The secondary structure analysis using web-based software (PredictProtein) predicted that these cysteine residues of Gcd could form disulfide bonds. This prediction was consistent with our experimental evidence that three of them (C235, C275, and C578) were necessary for the interaction between DsbA1 and Gcd. Gcd exerts its function as a monomer, therefore, we assumed that these cysteine residues might form intramolecular disulfide bonds or interact with other periplasmic proteins to influence the function of Gcd [30]. Previous works showed that in *Vibrio cholerae*, the presence of the bile salts causes dimerization of the transmembrane transcription factor TcpP by inducing intermolecular disulfide bonds in its periplasmic domain [31]. In addition, DsbA could induce TcpP dimerization in the presence of taurocholate [32].

Mutation of the *gcd* gene caused a strong accumulation of 2,4-DAPG in *P. protegens* CHA0 [22]. The differential contribution of *gcd* to 2,4-DAPG production might be closely related to carbon source metabolism and antibiotic production. An in silico analysis indicated that the gene encoding gluconate dehydrogenase (Gad), which converts gluconic acids to 2-ketogluconate, is not found in the *P. fluorescens* 2P24 genome, whereas the functional genes encoding the Gad protein and the Gcd protein exist in the CHA0 genome, suggesting that the pathway of glucose catabolism in strain 2P24 is different from that in strain CHA0 [22]. In addition, the antibiotics produced by strain CHA0 include 2,4-DAPG, pyoluteorin (Plt), and pyrrolnitrin (Prn), and the production of 2,4-DAPG and Plt show mutual inhibition [33]. However, no Plt and Prn, but only 2,4-DAPG, could be detected in strain 2P24 [4].

Although we do not fully understand the molecular mechanism of how DsbA1 influences the disulfide bond formation of Gcd, it is clear that in the absence of *dsbA1*, the activity of Gcd was significantly improved. Further study is necessary to understand the mechanism by which the cysteine residues (C235, C275, and C578) influence the function of Gcd in *P. fluorescens* 2P24.

## Conclusions

*P. fluorescens* 2P24 is an effective biocontrol agent of soilborne plant diseases caused by phytopathogens, and the production of the antibiotic compound 2,4-DAPG is necessary for its biocontrol traits. In this study, our data showed that mutation of *dsbA1*, which encodes a protein disulfide oxidoreductase caused the accumulation of 2,4-DAPG in a GacA-independent manner. Further analysis indicated that DsbA1 negatively regulated the production of 2,4-DAPG by fine-tuning the function of glucose dehydrogenase Gcd and three cysteine residues (C235, C275, and C578) in Gcd were required for the interaction between DsbA1 and Gcd. These findings provide a new insight into 2,4-DAPG production in which DsbA1 influences the production of 2,4-DAPG by influencing the function of Gcd.

## Methods

### Bacterial strains, plasmids, and growth conditions

Bacterial strains and plasmids used in this study are listed in Additional file 2: Table S1. *Escherichia coli* was routinely grown in Lysogenic broth (LB) medium at 37 °C. *Pseudomonas fluorescens* was cultured in LB medium, KB (King’s B medium) [34], or ABM medium [35] at 28 °C. When necessary, growth media were supplemented with ampicillin (Ap) (50 μg/ml), kanamycin (Km) (50 μg/ml), tetracycline (Tet) (20 μg/ml), and 5-bromo-4-chloro-3-indolyl-β-D-galactopyranoside (X-gal) (40 μg/ml).

### DNA techniques

Preparation of genomic DNA of *P. fluorescens*, plasmid DNA extraction, and other molecular assays were carried out using standard methods [36]. Electroporation of fresh *Pseudomonas* cells with plasmid DNA was performed as described previously [37]. Nucleotide sequences were determined on an ABI-Prism 373 automatic sequencer (Applied Biosystems). Nucleotide and deduced amino acid sequences were analyzed using a BLAST algorithm [38].

### Construction of strains and plasmids

To generate *P. fluorescens dsbA1*, *dsbA2*, *dsbB1*, *dsbB2*, and *gcd* mutants, homologous recombination procedures were performed using p2P24Km derivatives as described previously [17] (Additional file 2: Table S1). Plasmid pBBR1MCS-2 was used to restore the function of *dsbA1*, *dsbB1*, *dsbB2*, and *gcd* genes in the *dsbA1* mutant, the *dsbB1* mutant, the *dsbB2* mutant, and the *gcd* mutant, respectively. These four genes were amplified from the *P. fluorescens* 2P24 genome DNA by PCR using primers dsbA1–1/dsbA1–2, dsbB1–1/dsbB1–2, dsbB2–1/dsbB2–2, and gcd-BamHIF/gcd-SacIR (Additional file 2: Table S1). The PCR products were digested and cloned into pBBR1MCS-2 to generate pBBR-dsbA1, pBBR-dsbB1, pBBR-dsbB2, and pBBR-gcd, respectively.

### Site-directed mutagenesis of the Cys residues in the Gcd protein

To change the Cys residues into Ser residues in the Gcd protein, oligonucleotides containing single nucleotide substitutions were constructed and used the Fast Mutagenesis system (TransGen, Beijing, China) (Additional file 2: Table S1). The specificity of the nucleotide sequence was confirmed by DNA sequencing.

### β-Galactosidase assays

β-Galactosidase activities were quantified using the Miller method [23]. *P. fluorescens* 2P24 and its derivatives were grown at 28 °C in 50-ml flasks containing 20 ml of LB medium with shaking at 200 rpm. Cultures were then sampled at indicated time points. Assays were performed in duplicate at least three times.

### Quantification of 2,4-DAPG

Quantification of 2,4-DAPG was performed according to a previously mentioned method [39]. Briefly, 5 ml overnight cultures of *P. fluorescens* were extracted twice with 2.5 ml of ethyl acetate. The extracts were dried and suspended in 100 μl of 100% methanol and a portion (10 μl) was then analyzed using HPLC.

### Phosphate-solubilizing assay

To determine the phosphate-solubilizing ability of strain 2P24 and its derivatives, 5-μl drops of bacterial suspensions were deposited on National Botanical Research Institute’s Phosphate (NBRIP) agar plates containing insoluble tricalcium phosphate. Solubilization halos were measured after 20 d of incubation at 28 °C, using ImageJ (https://imagej.nih.gov/ij/index.html). The experiment was performed three times, with eight replicates per treatment.

### Bacterial two-hybrid assay

A bacterial two-hybrid assay was conducted as described previously [40]. PCR fragments corresponding to *dsbA1* and *gcd* were cloned into the plasmids pUT18c and pKT25. To analyze the interaction of each construct, strain *E. coli* BTH101 cells containing both pUT18C-fusion and pKT25-fusion constructs were cultured at 28 °C for 16 h, and β-galactosidase activities were measured [23].

### Motility assay

Swimming motility was tested on LB plates with 0.3% agar. Overnight bacterial cultures were adjusted to OD_600_ = 1.0 for further motility test. Aliquots (2 μL) were dropped onto the swim agar plates and incubated for 16 h at 28 °C. Motility was then determined qualitatively by examining the circular turbid zone. The twitching motility assay performed on LB agar plates (1% agar) by stab inoculating bacteria through the agar to the bottom of the petri dish. After incubation for 48 h at 28 °C, the halo at the bottom of the plate was visualized using crystal violet (1% [wt/vol]) coloration.

### Western blot analysis

To measure the protein levels of RsmA-FLAG and RsmE-FLAG, *P. fluorescens* cells containing the FLAG tag were cultured in LB at 28 °C for 12 h and 1-ml samples were taken. Cells were then suspended in phosphate-buffered saline (PBS) buffer and lysed by sonication. The protein in crude lysates was quantified using the Bradford protein assay (TaKaRa). Total proteins were subjected to SDS-PAGE gel electrophoresis and transferred onto PVDF membrane (Millipore). Blots were washed with PBS containing 0.05% Tween-20 and probed with rabbit-anti-FLAG antibody (Cowin-Biotech, Beijing, China) as primary antibody and mouse-anti-RNAP antibody as the loading control. The resulting bots were incubated for 1 min in chemiluminescence (ECL) reagent using the eECL Western Blot kit (Cowin-Biotech, Beijing, China) and the proteins bands were detected on the X-ray film.

### Statistical analysis

All experiments were performed in triplicate. The data were analyzed and compared by performing two-sample independent t-tests using DPS v9.50 (http://www.dpsw.cn/dps_eng/index.html).

## Supplementary information

**Additional file 1 Figure S1.** The interaction of DsbA1 with RsmA, RsmE, PhlF, and OprF in vivo. The DsbA1, RsmA, RsmE, PhlF, and OprF were fused with the T25 and T18 domains of CyaA from Bordetella pertussis, respectively, and the T25, T18 fusion pairs were transformed into *E. coli* BTH101. Cultures were grown at 30 °C for 8 h and the β-galactosidase activities were then measured by Miller method (Miller, 1972). The experiments were performed in triplicate, and the mean values ± SD are indicated.

**Additional file 2 Table S1.** bacterial strains, plasmids, and primers used in this study **Table S2.** Inhibition of *R. solani* by strain 2P24 and its derivatives on PDA agar.

## Data Availability

The genome sequence of *Pseudomonas fluorescens* 2P24 has been submitted to GenBank with accession number CP025542. The datasets used and/or analyzed during this study available from the corresponding author on reasonable request.

## Abbreviations

2,4-DAPG: 2,4-diacetylphloroglucinol
Gad: Gluconate dehydrogenase
Gcd: Glucose dehydrogenase
GidA: Glucose-inhibited division protein A
MAPG: Monoacetylphloroglucinol
PG: Phloroglucinol
PGPR: Plant growth-promoting rhizobacteria
Plt: Pyoluteorin
Prn: Pyrrolnitrin
sRNA: Small regulatory RNA
TrmE: tRNA modification GTPase
Trx: Thioredoxin
Xcc: Xanthomonas campestris pv. campestris

## References

[1] Haas D, Défago G (2005). Biological control of soil-borne pathogens by fluorescent pseudomonads. Nat Rev Microbiol.

[2] Dubuis C, Keel C, Haas D (2007). Dialogues of root-colonizing biocontrol pseudomonads. Eur J Plant Pathol.

[3] Keel C, Schnider U, Maurhofer M, Voisard C, Laville J, Burger U (1992). Suppression of root diseases by *Pseudomonas fluorescens* CHA0: importance of the bacterial secondary metabolite 2,4-diacetylphloroglucinol. Mol Plant-Microbe Interact.

[4] Wei HL, Zhou HY, Zhang LQ, Wang Y, Tang WH (2004). Experimental evidence on the functional agent of 2,4-diacetylphloroglucinol in biocontrol activity of *Pseudomonas fluorescens* 2P24. Acta Microbiol Sin.

[5] Bangera MG, Thomashow LS (1999). Identification and characterization of a gene cluster for synthesis of the polyketide antibiotic 2,4-diacetylphloroglucinol from *Pseudomonas fluorescens* Q2-87. J Bacteriol.

[6] Achkar J, Xian M, Zhao H, Frost JW (2005). Biosynthesis of phloroglucinol. J Am Chem Soc.

[7] Hayashi A, Saitou H, Mori T, Matano I, Sugisaki H, Maruyama K (2012). Molecular and catalytic properties of monoacetylphoroglucinol acetyltransferase from *Pseudomonas* sp. YGJ3. Biosci Biotechnol Biochem.

[8] Abbas A, McGuire JE, Crowley D, Baysse C, Dow M, O’Gara F (2004). The putative permease PhlE of *Pseudomonas fluorescens* F113 has a role in 2,4-diacetyphloroglucinol resistance and in general stress tolerance. Microbiology..

[9] Abbas A, Morrissey JP, Marquez PC, Sheehan MM, Delany IR, O’Gara F (2002). Characterization of interactions between the transcriptional repressor PhlF and its binding site at the *phlA* promoter in *Pseudomonas fluorescens* F113. J Bacteriol.

[10] Bottiglieri M, Keel C (2006). Characterization of PhlG, a hydrolase that specifically degrades the antifungal compound 2,4-diacetylphloroglucinol in the biocontrol agent *Pseudomonas fluorescens* CHA0. Appl Environ Microbiol.

[11] Yan X, Yang R, Zhao RX, Han JT, Jia WJ, Li DY (2016). Transcriptional regulator PhlH modulates 2,4-diacetylphloroglucinol biosynthesis in response to the biosynthetic intermediate and end product. Appl Environ Microbiol.

[12] Heeb S, Haas D (2001). Regulatory roles of the GacS/GacA two-component system in plant-associated and other gram-negative Bacteria. Mol Plant-Microbe Interact.

[13] Schnider U, Keel C, Blumer C, Troxler J, Défago G, Haas D (1995). Amplification of the housekeeping sigma factor in *Pseudomonas fluorescens* CHA0 enhances antibiotic production and improves biocontrol abilities. J Bacteriol.

[14] Whistler CA, Corbell N, Sarniguet A, Ream W, Loper JE (1998). The two-component regulators GacS and GacA influence accumulation of the stationary-phase sigma factor σ^S^ and the stress response in *Pseudomonas fluorescens* Pf-5. J Bacteriol.

[15] Péchy-Tarr M, Bottiglieri M, Mathys S, Lejbolle KB, Schnider-Keel U, Maurhofer M, Keel C (2005). RpoN (σ^54^) controls production of antifungal compounds and biocontrol activity in *Pseudomonas fluorescens* CHA0. Mol Plant-Microbe Interact.

[16] Zhang W, Zhao Z, Zhang B, Wu X, Ren Z, Zhang L (2014). Posttranscriptional regulation of 2,4-diacetylphloroglucinol production by GidA and TrmE in *Pseudomonas fluorescens* 2P24. Appl Environ Microbiol.

[17] Zhang Y, Zhang Y, Zhang B, Wu X, Zhang L (2018). Effect of carbon sources on production of 2,4-diacetylphoroglucinol in *Pseudomonas fluorescens* 2P24. Acta Microbiol Sin.

[18] Wei HL, Wang Y, Zhang LQ, Tang WH (2004). Identification and characterization of biocontrol bacterial strain 2P24 and CPF-10. Acta Phytopathol Sin.

[19] Rietsch A, Beckwith J (1998). The genetics of disulfide bond metabolism. Annu Rev Genet.

[20] Zhou YP, Wu XG, Zhou HY, He YQ, Zhang LQ (2010). Effect of gene *phlF* on 2,4-diacetylphloroglucinol production in *Pseudomonas fluorescens* 2P24. Acta Phytopathol Sin.

[21] Li X, Gu GQ, Chen W, Gao LJ, Wu XH, Zhang LQ (2018). The outer membrane protein OprF and the sigma factor SigX regulate antibiotic production in *Pseudomonas fluorescens* 2P24. Microbiol Res.

[22] de Werra P, Péchy-Tarr M, Keel C, Maurhofer M (2009). Role of gluconic acid production in the regulation of biocontrol traits of *Pseudomonas fluorescens* CHA0. Appl Environ Microbiol.

[23] Miller JH (1972). Experiments in molecular genetics.

[24] Matsushita K, Ameyama M (1982). D-Glucose dehydrogenase from *Pseudomonas fluorescens*, membrane-bound. Methods Enzymol.

[25] Dailey FE, Berg HC (1993). Mutants in disulfide bond formation that disrupt flagellar assembly in *Escherichia coli*. Proc Natl Acad Sci U S A.

[26] Nakamoto H, Bardwell JC (2004). Catalysis of disulfide bond formation and isomerization in the *Escherichia coli* periplasm. Biochim Biophys Acta.

[27] Arts IS, Ball G, Leverrier P, Garvis S, Nicolaes V, Vertommen D (2013). Dissecting the machinery that introduces disulfide bonds in *Pseudomonas aeruginosa*. mBio.

[28] Jiang BL, Liu J, Chen LF, Ge YY, Hang XH, He YQ (2008). DsbB is required for the pathogenesis process of *Xanthomonas campestris* pv. *campestris*. Mol Plant-Microbe Interact.

[29] Yan Q, Wu XG, Wei HL, Wang HM, Zhang LQ (2009). Differential control of the PcoI/PcoR quorum-sensing system in *Pseudomonas fluorescens* 2P24 by sigma factor RpoS and the GacS/GacA two-component regulatory system. Microbiol Res.

[30] Cozier GE, Anthony C (1995). Structure of the quinoprotein glucose dehydrogenase of *Escherichia coli* modelled on that of methanol dehydrogenase from *Methylobacterium extorquens*. Biochem J.

[31] Yang M, Liu Z, Hughes C, Stern AM, Wang H, Zhong Z (2013). Bile salt-induced intermolecular disulfide bond formation activates *Vibrio cholera* virulence. Proc Natl Acad Sci U S A.

[32] Xue Y, Tu F, Shi M, Wu CQ, Ren G, Wang X (2016). Redox pathway sensing bile salts activates virulence gene expression in *Vibrio cholera*. Mol Microbiol.

[33] Clifford JC, Buchanan A, Vining O, Kidarsa TA, Chang JH, McPhail KL (2015). Phloroglucinol functions as an intracellular and intercellular chemical messenger influencing gene expression in *Pseudomonas protegens*. Environ Microbiol.

[34] King EO, Ward MK, Raney DE (1954). Two simple media for the demonstration of pyocyanin and fluorescein. J Lab Clin Med.

[35] Chilton MD, Currier TC, Farrand SK, Bendich AJ, Gordon MP, Nester EW (1974). *Agrobacterium tumefaciens* DNA and PS8 bacteriophage DNA not detected in crown gall tomors. Proc Natl Acad Sci U S A.

[36] Sambrook J, Fritsch EF, Maniatis T (1989). Molecular cloning: a laboratory manual.

[37] Choi KH, Kumar A, Schweizer HP (2006). A 10-min method for preparation of highly electrocompetent *Pseudomonas aeruginosa* cells: application for DNA fragment transfer between chromosomes and plasmid transformation. J Microbiol Methods.

[38] Altschul SF, Madden TL, Schaffer AA, Zhang J, Zhang Z, Miller W (1997). Gapped BLAST and PSI-BLAST: a new generation of protein database search programs. Nucleic Acids Res.

[39] Shanahan P, O’Sullivan DJ, Simpson P, Glennon JD, O’Gara F (1992). Isolation of 2,4-diacetylphloroglucinol from a fluorescent pseudomonad and investigation of physiological parameters influencing its production. Appl Environ Microbiol.

[40] Karimova G, Pidoux J, Ullmann A, Ladant D (1998). A bacterial two-hybrid system based on a reconstituted signal transduction pathway. Proc Natl Acad Sci U S A.

