# Supplementary figures and images for: The Oxidoreductase DsbA1 negatively influences 2,4-diacetylphloroglucinol biosynthesis by interfering the function of Gcd in Pseudomonas fluorescens 2P24
Source: BMC Microbiol. 2020 Feb 24;20:39. doi: 10.1186/s12866-020-1714-1 (PMC7041245; doi:10.1186/s12866-020-1714-1)

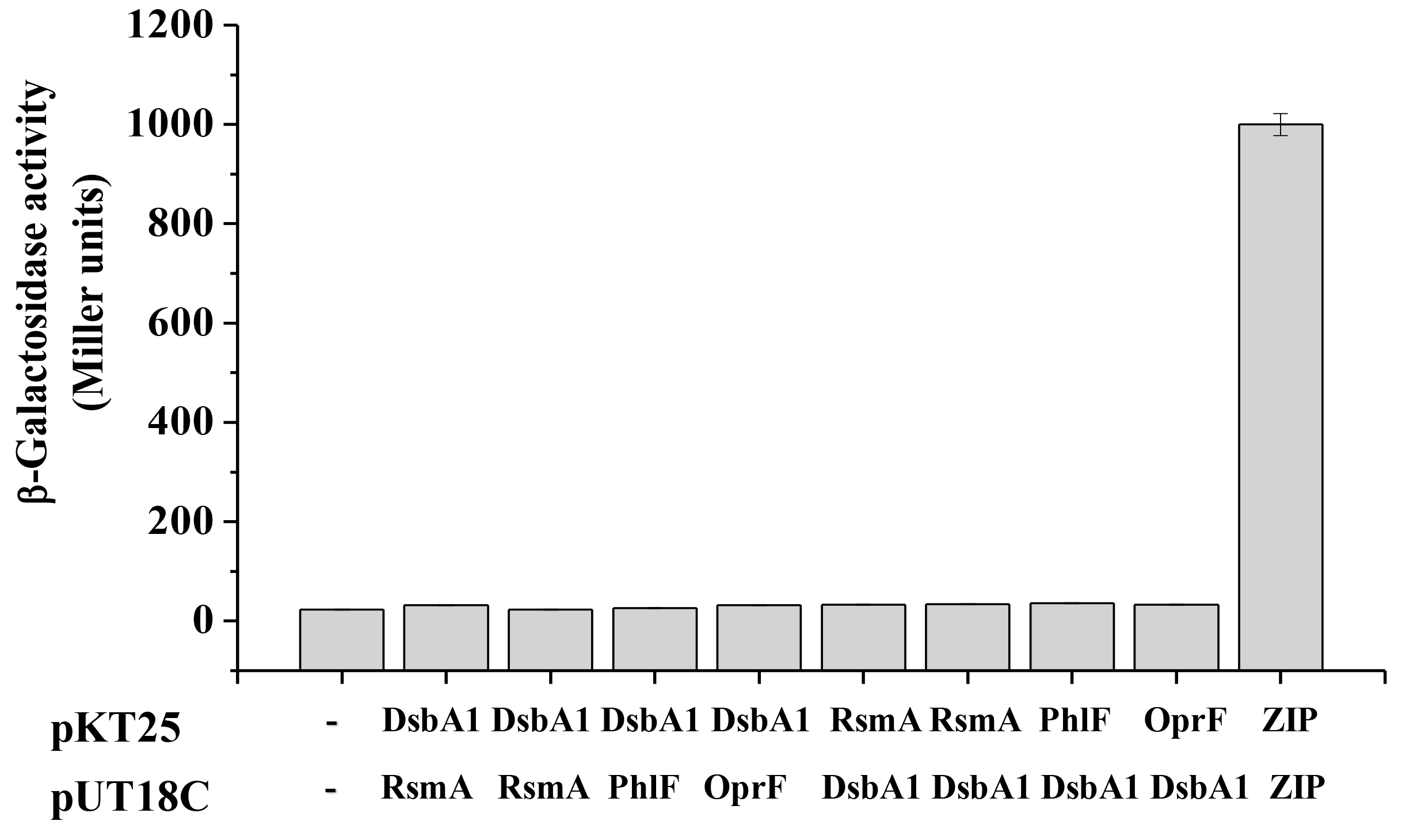

Supplement: Supplementary file 1 — Additional file 1 Figure S1. The interaction of DsbA1 with RsmA, RsmE, PhlF, and OprF in vivo. The DsbA1, RsmA, RsmE, PhlF, and OprF were fused with the T25 and T18 domains of CyaA from Bordetella pertussis, respectively, and the T25, T18 fusion pairs were transformed into E. coli BTH101. Cultures were grown at 30 °C for 8 h and the β-galactosidase activities were then measured by Miller method (Miller, 1972). The experiments were performed in triplicate, and the mean values ± SD are indicated. [file 12866_2020_1714_MOESM1_ESM.tif]
