# Supplementary material for: The Oxidoreductase DsbA1 negatively influences 2,4-diacetylphloroglucinol biosynthesis by interfering the function of Gcd in Pseudomonas fluorescens 2P24
Source: BMC Microbiol. 2020 Feb 24;20:39. doi: 10.1186/s12866-020-1714-1 (PMC7041245; doi:10.1186/s12866-020-1714-1)
Supplement: Supplementary file 2 — Additional file 2 Table S1. bacterial strains, plasmids, and primers used in this study Table S2. Inhibition of R. solani by strain 2P24 and its derivatives on PDA agar. [file 12866_2020_1714_MOESM2_ESM.docx]

**Table S1 bacterial strains, plasmids, and primers used in this study**

| Strains or plasmids | Relevant characteristics | Reference or source |
| --- | --- | --- |
| Strains |  |  |
| *Pseudomonas fluorescens* |  |  |
| 2P24 | Wild-type, Ap^r^ | Wei et al., 2004 |
| WPM20 | *dsbA1* deletion mutant, Ap^r^ | This work |
| WPM21 | *dsbA2* deletion mutant, Ap^r^ | This work |
| WPM22 | *dsbB1* deletion mutant, Ap^r^ | This work |
| WPM23  WPM24 | *dsbB2* deletion mutant, Ap^r^  *dsbB1* *dsbB2* double mutant, Ap^r^ | This work  This work |
| WPM25 | *gcd* deletion mutant, Ap^r^ | This work |
| WPM26 | 2P24 with a FLAG epitope sequence tagged to the C terminus of RsmA, Ap^r^ | Lab stock |
| WPM27 | 2P24 with a FLAG epitope sequence tagged to the C terminus of RsmE, Ap^r^ | Lab stock |
| WPM28 | WPM20 with a FLAG epitope sequence tagged to the C terminus of RsmA, Ap^r^ | This work |
| WPM29 | WPM20 with a FLAG epitope sequence tagged to the C terminus of RsmE, Ap^r^ | This work |
| PM203 | *gacA* deletion mutant, Ap^r^ | Yan et al., 2009 |
| WPM30 | *dsbA1 gacA* double mutant, Ap^r^ | This work |
| X-2 | Derivative of PM203, *dsbA1*::Tn*5*, Km^r^ | This work |
| *E. coli* DH5α | *supE44* *lacU*169 (*φ*80lacZ M15) *hsdR*17 *recA1* *endA1* *gyrA96 thi-1 relA1* | Sambrook et al., 1989 |
| BTH101 | *F-, cya-99, araD139, galE15, galK16, rpsL1 (Strr), hsdR2, mcrA1, mcrB1* | Euromedex |
| Plasmids |  |  |
| p6013-phlA | *phlA*′-′*lacZ* translational fusion, Tet^r^ | Zhang et al., 2018 |
| p2P24Km | Suicide plasmid with *sacB* used for homologous recombination, Km^r^ | Zhang et al., 2018 |
| p2P24Km-dsbA1 | p2P24Km with a deleted *dsbA1* gene, Km^r^ | This work |
| p2P24Km-dsbA2 | p2P24Km with a deleted *dsbA2* gene, Km^r^ | This work |
| p2P24Km-dsbB1 | p2P24Km with a deleted *dsbB1* gene, Km^r^ | This work |
| p2P24Km-dsbB1 | p2P24Km with a deleted *dsbB2* gene, Km^r^ | This work |
| p2P24Km-gcd | p2P24Km with a deleted *gcd* gene, Km^r^ | This work |
| pBBR1MCS-2 | Broad-host-range cloning vector, Km^r^ | Kovach et al., 1995 |
| pBBR-dsbA1 | pBBR1MCS-2 containing the *dsbA1* gene, Km^r^ | This work |
| pBBR-dsbB1 | pBBR1MCS-2 containing the *dsbB1* gene, Km^r^ | This work |
| pBBR-dsbB2 | pBBR1MCS-2 containing the *dsbB2* gene, Km^r^ | This work |
| pBBR-gcd | pBBR1MCS-2 containing the *gcd* gene, Km^r^ | This work |
| pBBR-gcdC235S | pBBR1MCS-2 containing gcd^C235S^, Km^r^ | This work |
| pBBR-gcdC275S | pBBR1MCS-2 containing gcd^C275S^, Km^r^ | This work |
| pBBR-gcdC578S | pBBR1MCS-2 containing gcd^C578S^, Km^r^ | This work |
| pBBR-gcdC306S | pBBR1MCS-2 containing gcd^C306S^, Km^r^ | This work |
| pBBR-gcdC330S | pBBR1MCS-2 containing gcd^C330S^, Km^r^ | This work |
| pBBR-gcdC678S | pBBR1MCS-2 containing gcd^C678S^, Km^r^ | This work |
| pKT25 | pSU40 derivative with T25 fragment of CyaA, Km^r^ | Euromedex |
| pUT18C | pUC19 derivative with T18 fragment of CyaA, C-terminal fusions, Ap^r^ | Euromedex |
| pKT25-dsbA1 | pKT25 containing the *dsbA1* gene, Km^r^ | This work |
| pUT18C-gcd | pUT18C containing the *gcd* gene, Ap^r^ | This work |
| pKT25-gcd | pKT25 containing the *gcd* gene, Km^r^ | This work |
| pUT18C-dsbA1 | pUT18C containing the *dsbA1* gene, Ap^r^ | This work |
| pKT25-gcdC235S | pKT25 containing *gcd*^C235S^, Ap^r^ | This work |
| pKT25-gcdC275S | pKT25 containing *gcd*^C275S^, Ap^r^ | This work |
| pKT25-gcdC306S | pKT25 containing *gcd*^C306S^, Ap^r^ | This work |
| pKT25-gcdC330S | pKT25 containing *gcd*^C330S^, Ap^r^ | This work |
| pKT25-gcdC578S | pKT25 containing *gcd*^C578S^, Ap^r^ | This work |
| pKT25-gcdC678S | pKT25 containing *gcd*^C678S^, Ap^r^ | This work |
| pUT18C-gcdC235S | pUT18C containing *gcd*^C235S^, Ap^r^ | This work |
| pUT18C-gcdC275S | pUT18C containing *gcd*^C275S^, Ap^r^ | This work |
| pUT18C-gcdC306S | pUT18C containing *gcd*^C306S^, Ap^r^ | This work |
| pUT18C-gcdC330S | pUT18C containing *gcd*^C330S^, Ap^r^ | This work |
| pUT18C-gcdC578S | pUT18C containing *gcd*^C578S^, Ap^r^ | This work |
| pUT18C-gcdC678S | pUT18C containing *gcd*^C678S^, Ap^r^ | This work |
| pKT25-ZIP | pKT25 derivative with the leucine zipper of GCN4, Km^r^ | Euromedex |
| PUT18C-ZIP | pUT18C derivative with the leucine zipper of GCN4, Ap^r^ | Euromedex |
|  |  |  |
| Primers | Sequence (5′→3′) | Amplicon |
| dsbA1-1 | TATCTAGACTCATTGTCCAG | *dsbA1* expression |
| dsbA1-2 | TAGAATTCGGGCCATGGGCGTCG |  |
| dsbB1-1 | TGTCTAGATATTACAGGAAGAAGTGCTTAATC | *dsbB1* expression |
| dsbB1-2 | ATGAATTCCAGCAGAGCCGTTACGAGTTCTTC |  |
| dsbB2-1 | ATGGTACCTCTTCGACCTGTCGGGAATTC | *dsbB2* expression |
| dsbB2-2 | ATGGTACCTCTTCGACCTGTCGGGAATTC |  |
| gcd-BamHIF | GCGGATCCTTCGCACGAAAAAGATGTAAAGC | *gcd expression* |
| gcd-SacIR | ACGAGCTCGCAAGGGTGTCGCCTTTTTCATG |  |
| pUT18C-gcdPstI | ACACTGCAGGATGAGCACTGAGGGTGCTTTGAG | BTH assay for cloning *gcd* gene in pUT18C |
| PUT18C-gcdBamHI | AGGGATCCTCATCCGGCAGCTTGTACGCGATC |  |
| gcdpKT25PstI | ATGCTGCAGGGATGAGCACTGAGGGTGCTTTG | BTH assay for cloning *gcd* gene in pKT25 |
| gcdpKT25BamHI2500 | TGGGATCCTCATCCGGCAGCTTGTACGCGATC |  |
| gcdBamHIF1 | ATGGATCCGAAATCACCTTGCTCACCGTACG | *gcd* deletion |
| 1KBgcdR1 | CATTCTGCTTCTATTGATGGGCCTGCCGATGACCTACACCGGCAAGGACG |  |
| 1KBgcdF2 | CGTCCTTGCCGGTGTAGGTCATCGGCAGGCCCATCAATAGAAGCAGAATG |  |
| gcdSacIR2 | ATGAGCTCGGGCCGGGGATTGGCGAGGCGTC |  |
| dsbB2-F1-BamHI | ATGGATCCGGCCGATCCTAGCCTGCTGCTG | *dsbB2 deletion* |
| dsbB2-R1 | CGACGTATCGCCGCGAAGCGGTCGTCGGCGATGGAAGCCATGAGAAACAA |  |
| dsbB2-F2 | TTGTTTCTCATGGCTTCCATCGCCGACGACCGCTTCGCGGCGATACGTCG |  |
| dsbB2-R2-HindIII | AGTAAGCTTCATCGCACAGGTCGTTGAACGCCA |  |
| pKT25dsbA1F | ACTCTAGAGATGCGTAATCTGATCACCAGCGC | BTH assay for cloning *dsbA1*gene in pKT25 |
| pKT25dsbA1R | GTGAATTCTTAGTTGGCAGCAGCCTTGTTGGCC |  |
| pUT18CdsbAR | TATGAATTCGAGTTGGCAGCAGCCTTGTTGGC | BTH assay for cloning *dsbA1*gene in pUT18C |
| dsbA1-BamHIF1 | ACGGATCCTCAAGGGCGGCATCCTGAAATAC | *dsbA1* deletion |
| dsbA1-R1 | CGATATTTGCCGTTGACGATCATGGTGGCGACGACGAGCGCGGCGCTGGT |  |
| dsbA1-F2 | ACCAGCGCCGCGCTCGTCGTCGCCACCATGATCGTCAACGGCAAATATCG |  |
| dsbA1-KpnIR2 | ATGGTACCAACGGCTGAGGGTGTCGCCGATG |  |
| dsbB1F-EcoRI1 | ATGAATTCGGTGCGGGCGATTGTAGTGAGCTGAC | *dsbB1* deletion |
| dsbB1R2 | TACATGCAGGTGGCCCTCGGTGAGGGTCTGTCCCTGGCGCAATGGGCGCT |  |
| dsbB1F3 | AGCGCCCATTGCGCCAGGGACAGACCCTCACCGAGGGCCACCTGCATGTA |  |
| dsbB1R-HindIII4 | TTAAAGCTTGAGCAGCAGAGCCGTTACGAGTTCTTC |  |
| gcdC235SF | TCAACGGCATGCTCTACGTGAGCACGCCCCAC | Mutation of Cys235 to Ser in *gcd* gene |
| gcdC235SR | TCACGTAGAGCATGCCGTTGACCTTCAGCGGG |  |
| gcdC275SF | GTTGGGCCCACATGACCAGCCGTGGCGTG | Mutation of Cys275 to Ser in *gcd* gene |
| gcdC275SR | TGGTCATGTGGGCCCAACCCTTGAAGTTC |  |
| gcdC306SF | CCGGCCAGCACCTCGAGCCCACGACGG | Mutation of Cys306 to Ser in *gcd* gene |
| gcdC306SR | TCGAGGTGCTGGCCGGCGTGGTGCTGG |  |
| gcdC330SF | CGACACCGGCAAGATGAGCGAAGACTTC | Mutation of Cys330 to Ser in *gcd* gene |
| gcdC330SR | TCATCTTGCCGGTGTCGGCGTTCAGGGC |  |
| gcdC578SF | GTTCGACCAGATGCTGAGCCGGATCGACT | Mutation of Cys578 to Ser in *gcd* gene |
| gcdC578SR | TCAGCATCTGGTCGAACGGGGTCACGC |  |
| gcdC678SF | GCCCATGGGCCTGCCTAGCCAGGCACCGG | Mutation of Cys678 to Ser in *gcd* gene |
| gcdC678SR | TAGGCAGGCCCATGGGCGAGAGCAGCGC |  |

^*^Ap, ampicillin; Km, kanamycin; Tet, tetracycline.

**References**

Kovach, M.E., Elzer, P.H., Hill, D.S., Robertson, G.T., Farris, M.A., Roop, R.M., et al. 1995. Four new derivatives of the broad-host-range cloning vector pBBR1MCS, carrying different antibiotic resistance cassettes. Gene. 166:175-176.

Sambrook, J., Fritsch, E.F., and Maniatis, T. 1989. Molecular Cloning: A Laboratory Manual, 2nd ed. Cold Spring Harbor Laboratory Press, Cold Spring Harbor, NY, USA.

Wei, H.L., Wang, Y., Zhang, L.Q., and Tang, W.H. 2004. Identification and characterization of biocontrol bacterial strain 2P24 and CPF-10. Acta Phytopathol. Sin. 34:80-85.

Yan, Q., Wu, X.G., Wei, H.L., Wang, H.M., and Zhang, L.Q. 2009. Differential control of the PcoI/PcoR quorum-sensing system in *Pseudomonas fluorescens* 2P24 by sigma factor RpoS and the GacS/GacA two-component regulatory system. Microbiol. Res. 164:18-26.

Zhang, Y., Zhang, Y., Zhang, B., Wu, X., and Zhang, L. 2018. Effect of carbon sources on production of 2,4-diacetylphoroglucinol in *Pseudomonas* *fluorescens* 2P24. Acta Microbiol. Sin. 58:1202-1212.

Table S2. Inhibition of *R. solani* by strain 2P24 and its derivatives on PDA agar.

| Strain | Inhibitory zone (cm) |
| --- | --- |
| 2P24 | 0.56 ± 0.05 |
| PM203 (*gacA*^-^) | 0 |
| X-1 | 0.21 ± 0.02 |
| X-2 | 0.23 ± 0.03 |
| X-3 | 0.25 ± 0.03 |
| X-4 | 0.29 ± 0.03 |

Figure S1. The interaction of DsbA1 with RsmA, RsmE, PhlF, and OprF *in vivo*. The DsbA1, RsmA, RsmE, PhlF, and OprF were fused with the T25 and T18 domains of CyaA from Bordetella pertussis, respectively, and the T25, T18 fusion pairs were transformed into *E. coli* BTH101. Cultures were grown at 30 ℃ for 8 h and the β-galactosidase activities were then measured by Miller method (Miller, 1972). The experiments were performed in triplicate, and the mean values ± SD are indicated.
